# Vitamin D‐Mediated Hypercalcemia in a Dog With Putative Cutaneous Pythiosis

**DOI:** 10.1002/ccr3.71594

**Published:** 2025-12-05

**Authors:** Jennifer Brodkin, Steven W. Frederick, Markus Rick, Peter S. Chapman

**Affiliations:** ^1^ BluePearl Pet Hospital Levittown Pennsylvania USA; ^2^ BluePearl Science Tampa Florida USA; ^3^ DiaSys Diagnostic Systems USA, LLC Wixom Michigan USA

**Keywords:** cutaneous pythiosis, dog, hypercalcemia, *Lagenidium*, oomycosis, *Pythium insidiosum*, vitamin D‐mediated

## Abstract

A 12‐month‐old dog was diagnosed with cutaneous pythiosis after presenting with a nonhealing metatarsal wound, an inguinal mass, and regional lymphadenopathy. Both cytology of the abnormal lymph nodes and histopathology of the inguinal mass showed granulomatous inflammation. ELISA serology was positive for *Pythium insidiosum.* The dog developed hypercalcemia secondary to excessive vitamin D production. This conclusion was based on the combination of hyperphosphatemia, suppressed parathyroid hormone, suppressed 25‐hydroxyvitamin D level, and a calcitriol level at the high end of the reference interval. This case report describes the protracted diagnosis of vitamin D‐mediated hypercalcemia in conjunction with cutaneous pythiosis infection. As this was not a known risk in pythiosis cases, delayed diagnosis and treatment of hypercalcemia, along with delayed diagnosis of the pythiosis infection and use of compounded itraconazole, contributed to a poor outcome.

## Summary

Periodic monitoring of calcium levels is recommended in dogs with pythiosis, and vitamin D‐mediated hypercalcemia should be considered when hypercalcemia is diagnosed in these cases.

## 1 Introduction

*Pythium insidiosum* is a water mold in the class Oomycetes [1, 2]. It is most reported in horses and young, large breed dogs [2, 3], while less commonly reported in cats, sheep, cattle, and exotic species [1, 2]. It is more prevalent in tropical and subtropical climates, though it is seen in other regions as well [2, 3, 4]. Within the United States in particular, it is most often reported in Southern states with only occasional cases seen in neighboring states [5]. Gastrointestinal and cutaneous pythiosis are the two most common clinical syndromes reported [2, 3], with the gastrointestinal form being more common in dogs [1]. Cutaneous pythiosis is characterized by nonhealing wounds and invasive masses with draining nodules and tracts. It most often occurs on the extremities, ventral neck, perineum, or at the base of the tail [2, 3, 4].

Hypercalcemia is reported in association with granulomatous inflammation caused by various infections, though the mechanism is not always clear [6]. One reported mechanism is inappropriate production of calcitriol by macrophages [7]. While there are multiple reports of vitamin D‐mediated, infection‐associated hypercalcemia in human medicine, reports in veterinary medicine are limited to an association with *Paecilomyses variotii, Cladophialophora bantiana*, and blastomycosis [8, 9, 10].

The objective of this case report is to describe vitamin D‐mediated hypercalcemia in a dog with cutaneous pythiosis. To the authors' knowledge, this is the first report of pythiosis in either veterinary medicine or human medicine where the mechanism of hypercalcemia is documented to be due to excessive vitamin D production.

## 2 Case History/Examination

Written informed consent was obtained from the pet's owner for this case report.

A 7‐month‐old castrated male golden retriever and standard poodle cross was presented to his primary veterinarian (day 0) for a left metatarsal wound that developed shortly after swimming in a creek. The dog was whelped and purchased in Pennsylvania and had only traveled between Pennsylvania and New Jersey since his owners acquired him. The dog was treated with a dexamethasone SP injection and oral cefpodoxime. Culture of the wound isolated methicillin‐resistant *Staphylococcus pseudintermedius* , and chloramphenicol was prescribed based on minimum inhibitory concentration results.

The metatarsal wound failed to heal and, on day 92, the dog was presented to a referral institution (separate from the authors' institution) for evaluation of the nonhealing wound, new metatarsal edema with extension to the inguinal region, and a newly identified enlarged inguinal lymph node.

## 3 Differential Diagnosis, Investigations, and Treatment

Comprehensive hematology and biochemistry panels at the time of evaluation on day 92 were within normal limits; total calcium was 10.2 mg/dL (reference interval [RI] 8.9–11.4 mg/dL). Cytology (Antech Diagnostics, Fountain Valley, CA) obtained from the enlarged inguinal lymph node showed pyogranulomatous inflammation with moderate numbers of neutrophils and macrophages (some multinucleated) and fewer numbers of lymphoid cells. No infectious agents were seen. A Tru‐Cut biopsy was performed on the edematous metatarsal region, and the histopathology (Antech Diagnostics) revealed pyogranulomatous inflammation as well. Routine H&E stain did not show microorganisms, and AFB stain for mycobacteria was negative. A few hyphae could be seen with PAS and GMS stains. *Pythium insidiosum* or other oomycotic infections were not suspected at the time since the hyphae stained with PAS. Fungal culture was negative; however, the dog was prescribed generic itraconazole (6 mg/kg, PO q12h, Sandoz Pharmaceuticals, Basel, Switzerland) regardless due to the presence of hyphae. Given financial constraints, the dog was transitioned to a compounded formulation at the same dosage a few days later.

Over the next 74 days, the dog developed a new, progressively enlarging inguinal mass that was causing lameness and dysuria. The dog was again presented to his primary veterinarian on day 166 for surgical debulking of the inguinal mass. Biopsies were submitted to a different laboratory than was used for the previous samples (IDEXX Laboratories, Westbrook, ME). Histopathology showed severe multinodular to coalescing inflammation and granulation tissue with fibrosis. Inflammation consisted of mostly epithelioid macrophages and multinucleated giant cells. There was evidence of distinct granuloma formation with areas of necrosis. Within the inflamed areas, occasional hyphal to round structures were identified. Unspecified special staining revealed irregular branching hyphae that were rarely septate. The pathologist considered this consistent with zygomycosis, though a specific species could not be determined based on histopathology alone. Biochemistry panel at that time showed a total hypercalcemia of 13.6 mg/dL (RI 8.2–12.4 mg/dL), a normal phosphorus of 5.3 mg/dL (RI 2.1–6.3 mg/dL), a minimally elevated BUN of 28 (RI 7–27 mg/dL), and a normal creatinine of 1.3 (RI 0.4–1.8 mg/dL). All other values were within normal limits.

## 4 Conclusion and Results

On day 171, the dog was presented to the authors' referral institution for evaluation of progressive inguinal swelling and lethargy. The metatarsal wound was reported to be progressing in size. The dog was still receiving compounded itraconazole at the time of evaluation. A complete biochemistry profile and hematology panel was repeated. Abnormalities included an ionized hypercalcemia of 2.19 mmol/L (RI 1.12–1.42 mmol/L), total hypercalcemia of > 15.3 mg/dL (RI 9.0–12.2 mg/dL), hyperphosphatemia of 7.0 mg/dL (RI 1.9–5.0 mg/dL), hyperglobulinemia of 4.7 g/dL (RI 2.0–3.6 g/dL), hypercholesterolemia of 390 mg/dL (RI 120–310 mg/dL), normal albumin of 2.7 g/dL (RI 2.5–4.0 g/dL), normocytic normochromic regenerative anemia with hematocrit of 17% (RI 36%–60%) and reticulocyte count of 76.8 × 10^3^/μL (RI < 60 × 10^3^/μL), neutrophilia with a neutrophil count of 24.0 × 10^3^/μL (RI 2.1 × 10^3^/μL—10.6 × 10^3^/μL) and 0.0 bands (RI 0/μL—300/μL), and a monocytosis of 3.0 × 10^3^/μL (RI 0/μL—840/μL). All other values were within normal limits. Thoracic radiographs were unremarkable. Abdominal ultrasound showed a large, heterogenous, soft tissue mass at the central inguinal region causing displacement of the os penis ventrally, an echogenic fluid pocket at the caudal left aspect of this mass, as well as moderate–severe, hypoechoic regional (medial iliac) lymphadenopathy. This fluid pocket was aspirated and clinician review of cytology showed sheets of degenerate neutrophils with a mixed morphology population of intracellular bacteria. The dog was administered a packed red blood cell transfusion (7.5 mL/kg over 4 h) and a pamidronate infusion (1.5 mg/kg over 2 h). The dog's ionized calcium decreased to 1.97 mmol/L (RI 1.12–1.42 mmol/L) after 10 h but then increased to 2.09 mmol/L (RI 1.12–1.42 mmol/L) after an additional 6 h. The owners elected humane euthanasia on day 173.

Following euthanasia, additional diagnostic results were reported. ELISA serology for anti‐*Pythium insidiosum* antibody was performed through the Louisiana State University Pythium Lab. This was positive for *Pythium insidiosum* seroreactivity at a percent positivity of 67%. Percent positivity in dogs with active pythiosis ranges from 40% to 100% versus 5% to 10% in healthy dogs. Parathyroid hormone was low‐normal (0.50 pmol/L, RI 0.50–5.80 pmol/L), parathyroid hormone‐related protein was undetectable (0.0 pmol/L, RI 0.0–1.0 pmol/L), 25‐hydroxyvitamin D was low (28 nmol/L, RI 60.0–215.0 nmol/L), and calcitriol was high‐normal (509 pmol/L, RI 164–523 pmol/L).

## 5 Discussion

The case described in this report is the first instance of vitamin D‐mediated hypercalcemia associated with pythiosis, another oomycete infection, or fungal infection in human or veterinary medicine. In veterinary medicine, reports of vitamin D‐mediated hypercalcemia associated with fungal disease are limited to a dog with disseminated *Paecilomyses variotii* infection [10], a dog with abdominal *Cladophialophora bantiana* [8], and a cat with disseminated blastomycosis [9]. In humans, it has been documented in association with cryptococcus [11, 12, 13], coccidiomycosis [11], disseminated *Candidia albicans* [14], and *Pneumocystis carinii* [15] fungal infections. Vitamin D‐mediated hypercalcemia associated with oomycete infection has not been reported in either veterinary or human medical literature.

Hypercalcemia of unknown etiology was reported in a dog with gastric pythiosis [16]. In that case, parathyroid hormone was at the low end of the reference interval, parathyroid hormone‐related protein was not detectable, 25‐hydroxyvitamin D was below the lower reference interval, and calcitriol was not measured. The mechanism for the hypercalcemia was not identified in that case, but it was presumed to be due to the *Pythium insidiosum* infection given that hypercalcemia resolved with resection of the gastric lesion. Hypervitaminosis D could not be definitively ruled out because calcitriol levels were not measured until 8 days following surgical resection, at which point the calcium level had normalized [16]. Hypercalcemia was also reported in two dogs in California with gastrointestinal pythiosis, but the mechanism was not investigated in those cases [17, 18]. In the case described in this report, the dog's hypercalcemia was presumed to be mediated by vitamin D due to the combination of hyperphosphatemia, suppressed parathyroid hormone, suppressed 25‐hydroxyvitamin D level, and a calcitriol level at the high end of the reference interval. Vitamin D‐mediated hypercalcemia associated with fungal infections is due to the granulomatous inflammation rather than to the fungus itself. One reported mechanism is inappropriate calcitriol production by macrophages [7, 12, 19]. Calcitriol is overproduced due to 1‐α‐hydroxylation of 25‐hydroxyvitamin D within macrophages [13, 20, 21]. This leads to increased intestinal absorption of calcium and enhanced bone resorption [12, 20]. Calcitriol is the biologically active form of vitamin D [6], so we would expect this to be suppressed if the hypercalcemia in this dog was due to a non‐vitamin D‐mediated mechanism. The dog's low 25‐hydroxyvitamin D level rules out exogenous vitamin D toxicity, such as with ingestion of cholecalciferol rodenticide, as this would result in a significantly elevated 25‐hydroxyvitamin D, suppressed parathyroid hormone, and down regulation of calcitriol via inhibition of 1‐α‐hydroxylase activity and enhancement of 24‐hydroxylase activity [6, 22]. There was also no history of exposure to topical vitamin D analogs or supplements that could have resulted in vitamin D toxicity.

Successful treatment of pythiosis generally requires surgical resection of all infected tissue with wide margins, which may necessitate limb amputation for cutaneous pythiosis [1, 2, 3, 4]. While nonresectable pythiosis carries a poor prognosis, there is a report of colonic pythiosis being successfully managed in three dogs using a combination of itraconazole, terbinafine, and prednisone [18]. The addition of an anti‐inflammatory dose of corticosteroids can be helpful in these non‐surgical cases as the inflammatory response is thought to contribute to disease severity [2, 18]. However, this positive outcome with the addition of corticosteroids has only been documented for cases of gastrointestinal pythiosis and not cutaneous. Assuming surgical excision is pursued, postoperative antifungal therapy with itraconazole and terbinafine is often required if wide margins cannot be achieved [2, 3]. Multiple studies have demonstrated poor bioavailability of compounded itraconazole when compared to generic and innovator‐formulated versions [23, 24]. The use of compounded itraconazole likely contributed to treatment failure in this dog. In addition to traditional therapy, mefenoxam is an agricultural fungicide that has been shown to inhibit the growth of *P. insidiosum* in vitro and has shown benefit as part of a multiagent treatment protocol in dogs. However, further investigation is required to determine the oral dose needed to reach adequate tissue levels [4]. An immunotherapy product derived from *P. insidiosum* antigens has also been used to treat people and horses with pythiosis, though efficacy has been poor in dogs [2].

In human medicine, vitamin D‐mediated hypercalcemia is managed with reduction of sunlight exposure and dietary calcium intake [13, 21]. Corticosteroids are also used because they inhibit 1,25‐dihydroxyvitamin D‐mediated intestinal absorption of calcium [13, 21, 25]. Corticosteroids, ketoconazole, hydroxychloroquine, and chloroquine have been shown to reduce 1,25‐dihydroxyvitamin D levels by acting directly on macrophages to inhibit extra‐renal 1‐α‐hydroxylation in patients with granulomatous inflammation from sarcoidosis [21, 26]. Given this, as well as the previous report of positive outcomes seen for gastrointestinal pythiosis treated in part with corticosteroids [18], anti‐inflammatory doses of corticosteroids may be of benefit in cases of fungal or oomycotic disease with vitamin D‐mediated hypercalcemia. However, caution should be used because corticosteroids can lead to worse outcomes in cases of infectious disease [27]. Corticosteroid therapy was not utilized in this case due to the rapid progression and lack of definitive diagnosis at the time of humane euthanasia, and so it is unknown if it would have improved the outcome.

Extension to regional lymph nodes is common in cutaneous pythiosis [2, 3, 4]. Although it was not described as such on the biopsy report, the anatomic location of the inguinal lesion was consistent with that of the lymph node, which can be presumed to have been effaced by the granulomatous inflammation. The hyphae visualized on the metatarsal wound biopsy stained positive for PAS. This is less commonly seen for *P. insidiosum* [2, 3], but it does occur [1]. The appearance of the hyphae seen on the inguinal mass was described as representative of zygomycosis, though zygomycosis cannot be reliably differentiated from pythiosis based on cytology or histopathology alone [2, 3]. The biopsy samples and digital images were over 10 years old and thus discarded by the laboratory and could not be reviewed to further characterize hyphal morphology, which is a limitation of this case. However, serological testing has a high specificity [2, 3, 4] with a cut off of 40% showing 100% accuracy for differentiating pythiosis from lagenidiosis and fungal infections [28]. This case had a percent positivity of 67%. Given these results combined with the complete clinical picture, infection with *P. insidiosum* was the most likely cause of the skin lesions and hypercalcemia.

In conclusion, this is the first report of vitamin D‐mediated hypercalcemia in a dog with cutaneous pythiosis. There are three other reports of hypercalcemia associated with gastrointestinal pythiosis in dogs, but in those reports the mechanism of hypercalcemia was unknown [16, 17, 18]. Pythiosis should be considered in dogs presenting with hypercalcemia, especially if they reside within or have traveled to an endemic area. Calcium levels should be monitored in dogs with suspected pythiosis, and vitamin D‐mediated hypercalcemia should be considered when total serum calcium and ionized calcium are reported outside of the reference interval in these cases.

## Author Contributions

**Jennifer Brodkin:** data curation, formal analysis, investigation, writing – original draft, writing – review and editing. **Steven W. Frederick:** data curation, formal analysis, project administration, writing – original draft, writing – review and editing. **Markus Rick:** formal analysis, investigation, writing – review and editing. **Peter S. Chapman:** conceptualization, data curation, formal analysis, investigation, methodology, writing – review and editing.

## Funding

The authors have nothing to report.

## Ethics Statement

The authors have nothing to report.

## Consent

Written informed consent from the pet's owner was obtained for this case report.

## Conflicts of Interest

The authors declare no conflicts of interest.

## Data Availability

Data sharing not applicable to this article as no datasets were generated or analysed during the current study.
